# Trends in Irritable Bowel Syndrome Incidence among Taiwanese Adults during 2003–2013: A Population-Based Study of Sex and Age Differences

**DOI:** 10.1371/journal.pone.0166922

**Published:** 2016-11-28

**Authors:** Chieh-Hsin Pan, Chun-Chao Chang, Chien-Tien Su, Pei-Shan Tsai

**Affiliations:** 1 School of Nursing, College of Nursing, Taipei Medical University, Taipei, Taiwan; 2 Department of Nursing, Taipei Medical University Hospital, Taipei, Taiwan; 3 Division of Gastroenterology and Hepatology, Department of Internal Medicine, School of Medicine, College of Medicine, Taipei Medical University, Taipei, Taiwan; 4 Division of Gastroenterology and Hepatology, Department of Internal Medicine, Taipei Medical University Hospital, Taipei, Taiwan; 5 School of Public Health, College of Public Health, Taipei Medical University, Taipei, Taiwan; 6 Division of Family Medicine, School of Medicine, College of Medicine, Taipei Medical University, Taipei, Taiwan; 7 Department of Nursing, Wan Fang Hospital, Taipei, Taiwan; 8 Sleep Research Center, Taipei Medical University Hospital, Taipei, Taiwan; University Hospital Llandough, UNITED KINGDOM

## Abstract

**Background:**

No population-based irritable bowel syndrome (IBS) incidence data among Taiwanese adults are available. Whether IBS is associated with risk of organic colonic diseases remains unanswered. We investigated 1) the sex- and age-stratified trends in the annual incidence of IBS, and 2) the risk of selected organic diseases in patients with IBS compared with those without IBS among Taiwanese adults during 2003–2013.

**Methods:**

Medical claims data for 1 million randomly selected beneficiaries were obtained and analyzed. Patients with IBS were considered eligible for enrollment if they aged between 20 and 100 and had at least two medical encounters with IBS codes within 1 year. To test whether there was a linear secular trend in IBS incidence over time, multivariate Poisson regression with generalized estimating equation model was conducted. The risk of selected organic diseases associated with IBS was examined using multivariate Cox proportional hazard regression.

**Results:**

From 2003 to 2013, the incidence of IBS significantly decreased over time [adjusted incidence rate ratio (IRR) = 0.97, p< 0.001]; the incidence of IBS significantly increased with age (adjusted IRR = 1.03, p < 0.001) and was significantly higher in women than in men (adjusted IRR = 1.14, p< 0.001). IBS significantly associated with increased risk of microscopic colitis, inflammatory bowel disease, and colorectal cancer during a 10-year follow-up period.

**Conclusions:**

The incidence of IBS increased with age and was slightly higher in women than in men among Taiwanese adults. During 2003–2013, IBS incidence gradually decreased over time. IBS may increase risk of several colonic organic diseases.

## Introduction

The global prevalence of irritable bowel syndrome (IBS) was estimated to be 11.2%[1]. The prevalence of IBS is significantly lower in Asian adults than in North American and Latin American adults, as reported by the Rome Foundation working team[2]; this suggests the existence of racial and ethnic variations in IBS incidence and prevalence. Population-based studies reporting national IBS incidence and prevalence rates in Asia are scant. Since the development of the Rome II diagnostic criteria in 1999[3] and that of the Rome III criteria in 2006[4], the diagnosis of functional gastrointestinal disorders has gained a consensus among gastroenterologists worldwide. In Taiwan, a survey of a population receiving physical check-ups at a medical center indicated that the prevalence rates of IBS, determined according to the Rome II and I criteria, were 22.1% and 17.5%, respectively[5], whereas a secondary analysis of nation-wide survey data demonstrated that the prevalence, determined according to the Rome III criteria, was 4.4%[6]. Thus far, no IBS incidence data in Taiwanese adults are available. Therefore, investigating the recent trends in the annual incidence of IBS among Taiwanese adults is crucial.

Reports on trends in the national incidence rates of IBS are of significance and interest to healthcare clinicians and health policymakers because such data not only clarify the societal and healthcare burdens of IBS but also facilitate decision making for healthcare resource and research dollar allocation. Therefore, conducting a population-based nationwide study to investigate the trends in the annual incidence of IBS among Taiwanese adults over the past decade (2003–2013) is imperative because no national data regarding this are available to date.

Regarding sex differences in IBS prevalence, IBS is more predominant in women[2, 7]. Female sex hormones have been suggested to contributed to this predominance[8, 9]. Although the effect of sex hormones on IBS symptoms remains inconsistent in the literature, studies have consistently reported a decline in the incidence rate of IBS after menopause[9]. A survey of a sample of Taiwanese adults receiving physical check-ups did not determine female predominance in the prevalence of IBS[10]. Accordingly, we investigated the sex- and age-stratified national trends in the annual incidence rates of IBS and incidence per 10 000 person-years in Taiwanese adults during 2003–2013.

Because IBS diagnosis is based on the presence of typical features and exclusion of selected organic diseases[4], several diseases must be considered in IBS patients including inflammatory bowel disease (IBD), microscopic colitis, celiac disease, and colorectal cancer[11]. Risk of IBDs (ulcerative colitis and Crohn disease)[12–14], celiac disease, and colorectal cancer[13] is higher in patients with IBS than in those without IBS. However, among 4528 patients who underwent diagnostic colonoscopy examinations at 7 centers in Japan, no differences in the prevalence of organic colonic diseases were noted between patients fulfilling and not fulfilling the Rome III criteria[15]. A prospective case–control trial in the United States revealed that the prevalence of structural abnormalities of the colon was similar between suspected nonconstipation IBS patients and healthy controls, as revealed through colonoscopy, despite the identification of microscopic colitis in a small proportion of patients with IBS symptoms[16]. To clarify further, we examined the risk of selected organic colonic diseases in patients with IBS compared with those without IBS during a 10-year follow-up period. We also explored the rate of use of endoscopic procedures (colonoscopy and sigmoidoscopy) in patients diagnosed with IBS in this study.

## Materials and Methods

### Data Source

We analyzed data retrieved from the Longitudinal Health Insurance Database 2010 (LHID2010) released by the Taiwan National Health Research Institutes. The LHID2010, a subset of the National Health Insurance Research Database, includes all medical claims data and registration files from 1996 to 2013 for 1 million enrollees randomly selected from the 2010 Registry for Beneficiaries (n = 27.38 million) of the Taiwan National Health Insurance program. The Taiwan National Health Research Institutes reported that no statistically significant differences exist in the distribution of sex, age, or average insured payroll-related amount between the LHID2010 and National Health Insurance Research Database beneficiaries.

### Study Samples

#### IBS incidence rate

To investigate the sex- and age-stratified national trends in the annual incidence rates of IBS and incidence per 10 000 person-years in Taiwanese adults, patients with IBS were considered eligible for enrollment if they were aged 20–100 years and had at least 2 medical encounters (either ambulatory care or hospitalization) within 1 year with a diagnosis corresponding to the International Classification of Disease, Ninth Revision, Clinical Modification (ICD-9-CM) code of IBS (564.1) during 2003–2013. Patients with missing sex information and patients with a diagnosis of IBS before 2003 were excluded. For incidence rate calculation, we excluded cases being diagnosed already with IBS in earlier years, and we thus included only incident cases.

#### Risk of organic disease associated with IBS

To examine the risk of selected organic colonic diseases in patients with IBS compared with those without IBS, we tracked a cohort of IBS incident cases identified in the year of 2003 and an age- and sex-matched non-IBS cohort during a 10-year follow-up period. Patients who received a new diagnosis of IBS on 2 separate visits during 2003 (IBS group) and age- and sex-matched individuals without a diagnosis of IBS (non-IBS group) among the 2003 beneficiaries in the LHID2010 were identified (IBS and non-IBS matched at 1:4 ratio). Because we included only incident IBS cases in 2003, patients who had a diagnosis of IBS before 2003 (n = 3197) were excluded from the IBS group. Patients were assigned to the non-IBS group if they had no IBS diagnosis before, during, or after 2003 (n = 113 034). Consequently, patients who had only one diagnosis of IBS (ICD-9-CM code 564.1) during 2003 (n = 6664) were excluded. In addition, those who with prior history of any organic disease before the index date (n = 1157) were also excluded from both groups. Finally, 1225 patients with IBS and 4900 age- and sex-matched individuals without IBS were included.

### Demographics

Demographic data considered in this study included age, sex, insurance premium, and urbanization level; the data were obtained from the beneficiary records of the LHID2010. For the age-specific incidence rates of IBS, the enrolled patients were stratified into 6 strata: 20–29, 30–39, 40–49, 50–59, 60–69, and 70–99 for both sexes.

### Organic Disease as Endpoints

Celiac disease, microscopic colitis, IBDs (ulcerative colitis and Crohn disease), and colorectal cancer were selected as endpoints and tracked from the index date to the end of 2013 in patients who received a new diagnosis of IBS on 2 separate visits during 2003 and age- and sex-matched individuals without a diagnosis of IBS among the 2003 beneficiaries in the LHID2010. Patients who had experienced any one of the five endpoints were considered to have experienced the composite outcome.

### Colonoscopy and Sigmoidoscopy Before IBS Diagnosis

Colonoscopy and sigmoidoscopy use within 2 years (730 d) before the second diagnosis of IBS (ICD-9-CM code 564.1) was identified through ICD-9-CM codes of procedures (451, 4511–4516, 4519, 4523, and 4524) or orders (28017C, 49014C, 49025C, 49027C, and 28013C).

### Statistical Analyses

The annual crude incidence rates of IBS were estimated by dividing the number of incidence cases of IBS by the total population in the LHID2010 during 2003–2013. By using the World Health Organization 2000 standard population, we also calculated age-standardized IBS incidence rates from 2003 to 2013. Furthermore, the incidence of IBS per 10 000 person-years from 2003 to 2013 was estimated from the 2003 beneficiaries in the LHID2010. These analyses were all stratified by sex and age groups and comparisons between men and women in incidence of IBS in a specific age group were performed by Cox proportional hazard regression models.

To test for a linear secular trend in IBS incidence over the study period (2003–2013), the Poisson regression with a generalized estimating equation (GEE) model was performed using the calendar year as the independent variable. To account for the confounding effect of socioeconomic status and identify the independent age and sex effects, we performed multivariate Poisson regression GEE analyses with adjustments for insurance premium and urbanization level.

Differences in the incidence rates of selected organic diseases were first tested by chi-square test. We then examined the hazard ratios (HRs) and the associated 95% confidence intervals (CIs) of selected organic diseases including celiac disease, microscopic colitis, IBDs, and colorectal cancer associated with IBS by using multivariate Cox proportional hazard regression, with IBS disease status serving as the independent variable and the presence of the composite outcome of organic diseases as well as the presence of each organic disease serving as the dependent variable in separate models after adjustments for age, sex, insurance premium, and urbanization level. In addition, separate analyses for men and women were performed to determine the associations of IBS with the organic diseases.

The rates of documented endoscopy use were calculated through procedure or order ICD-9-CM codes within 2 years before the second diagnosis of IBS (ICD-9-CM code 564.1) in patients with incident IBS and then stratified by sex and age groups.

## Ethical Considerations

We obtained data from the LHID2010, which contains anonymous data from 1 million randomly selected beneficiaries. All information that can be used to identify patients, healthcare providers, or medical institutions involved is scrambled before entry into the National Health Insurance Database and further encoded by the National Health Research Institutes before releasing the Longitudinal Health Insurance Database 2010 to researchers. Our study complied with the Personal Information Protection Act of Taiwan and was exempted from full review by the Joint Institutional Review Board of Taipei Medical University (TMU-JIRB No.:N201605038).

## Results

### IBS Incidence Rate

The annual incidence rates of IBS from 2003 to 2013 are presented in Table 1. The crude incidence rates of IBS fluctuated between 2003 and 2007, decreased considerably in 2008, and finally decreased gradually to 47.08 per 10 000 person-year in 2013, showing a 12.53% drop. The age-adjusted incidence of IBS revealed a 20.81% reduction in the incidence of IBS from 2003 (56.28 per 10 000 person-years) to 2013 (44.56 per 10 000 person-years).

**Table 1:** Trends in irritable bowel syndrome (IBS) incidence rates in Taiwanese adults during 2003–2013 (N = 7 634 281).

Incidence rate	Incidence per 10 000 person-y											
	2003	2004	2005	2006	2007	2008	2009	2010	2011	2012	2013	Change (%)
Crude	53.83	56.86	52.62	53.00	53.90	47.95	49.41	47.89	46.97	48.66	47.08	−12.53
Male	50.23	53.91	49.15	50.34	52.30	45.94	46.99	43.93	43.20	46.40	43.94	−12.52
Female	57.23	59.66	55.90	55.53	55.40	49.83	51.69	51.62	50.50	50.78	50.04	−12.58
Adjusted ^a^	56.28	58.19	53.38	53.43	53.42	47.10	48.14	46.24	44.62	45.89	44.56	−20.81
Male	52.05	55.03	49.61	50.71	51.26	44.90	45.48	42.07	40.55	43.48	41.28	−20.69
Female	60.05	61.14	56.90	56.03	55.37	49.22	50.72	50.22	48.41	48.23	47.67	−20.61

Change (%): percentage of changes in the incidence and prevalence rates of IBS between 2003 and 2013.

^a^ Age-adjusted based on the World Health Organization 2000 standard population.

**Fig 1:**
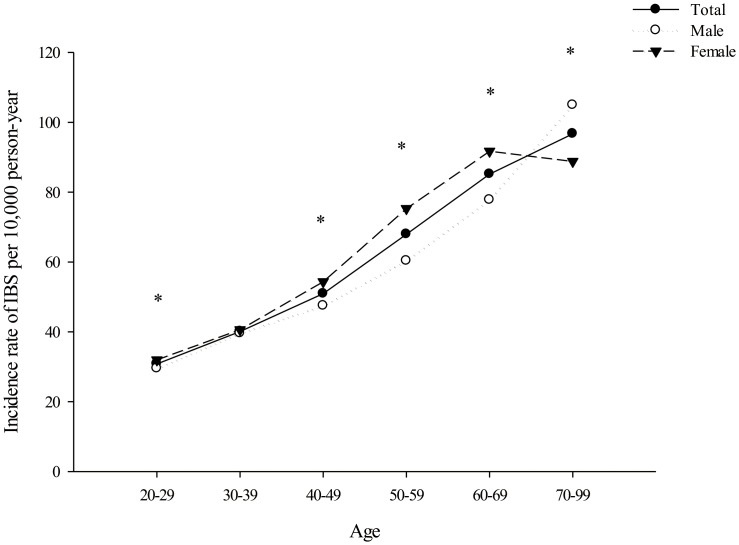
Age- and sex-stratified incidence rates of irritable bowel syndrome (IBS) per 10 000 person-years in Taiwan. Comparisons between men and women in incidence of IBS in a specific age group were performed by Cox proportional hazard regression models: P < 0.05.

During 2003–2013, the overall incidence of IBS per 10 000 person-years was 51.27, and the incidence in men and women was 48.90 and 53.51 per 10 000 person-years, respectively. The age- and sex-stratified incidence of IBS per 10 000 person-years is shown in Fig 1: Women showed a higher IBS incidence rate than men did in all age groups, except for the 30–39 (incidence rates were similar in both sexes) and 70–99 (incidence rate was higher in men than in women) age groups.

### Secular Trends in IBS Incidence

Multivariate Poisson regression GEE analyses showed that calendar year, age, and sex significantly predicted IBS incidence (Table 2) after adjustment for insurance premium and urbanization level. The IBS incidence rate decreased significantly over time (adjusted incidence rate ratio [IRR] = 0.97, *P* <.001); nevertheless, it increased with age (adjusted IRR = 1.03, *P* <.001), and it was significantly higher in women than in men (adjusted IRR = 1.14, *P* <.001).

**Table 2:** Factors predicting irritable bowel syndrome (IBS) incidence in Taiwan during 2003–2013 (N = 7 634 281).

Variables	Crude IRR	95% CI	*P* value ^a^	Adjusted IRR	95% CI	*P* value^b^^,^^c^
Year	0.98	0.98–0.99	<.001	0.97	0.97–0.97	<.001
Age	1.02	1.02–1.02	<.001	1.03	1.02–1.03	<.001
Female sex	1.12	1.10–1.14	<.001	1.14	1.12–1.16	<.001
Insurance premium (NTD)		–			–	
>=40000	0.95	0.92–0.98	<.001	1.15	1.12–1.19	<.001
20000–39999	0.97	0.95–0.99	0.007	1.09	1.06–1.11	<.001
<20000	1.00			1.00		
Urbanization ^c^						
Urban	0.89	0.86–0.92	<.001	1.05	1.01–1.09	0.017
Suburban	0.87	0.83–0.90	<.001	1.00	0.96–1.04	0.808
Rural	1.00			1.00		

IRR = incidence rate ration; CI = confidence interval.

^a^ Tested by univariate Poisson regression with GEE model.

^b^ Tested by multivariate Poisson regression GEE analysis.

^c^ With missing data for the urbanization level variable (n = 749 662).

### Association of IBS with Future Organic Colonic Disease Onset

The incidence rates of microscopic colitis (59.8% vs. 43.6%), IBD (7% vs. 3.8%), and colorectal cancer (4.6% vs. 1.3%) were significantly higher in the IBS group than in the non-IBS group. Celiac disease was nonexistent in the IBS group. Multivariate Cox proportional hazard regression revealed that the IBS group was associated with an increased risk of the composite outcome of organic diseases (adjusted HR = 1.77, 95% CI = 1.63–1.92), microscopic colitis (adjusted HR = 1.72, 95% CI = 1.58–1.87), IBD (adjusted HR = 1.92, 95% CI = 1.49–2.48), and colorectal cancer (adjusted HR = 3.63, 95% CI = 2.54–5.19), compared with the non-IBS group over the 10-year follow-up period (2003–2013), after adjustment for age, sex, insurance premium, and urbanization level (Tables 3 and 4). When men and women were analyzed separately, the association of IBS with microscopic colitis, IBD, and colorectal cancer remained significant in both groups after adjustment for age, insurance premium, and urbanization level (Table 5).

**Table 3:** Factors predicting risk of onset of the composite outcome of organic diseases.

Variables	HR	95% CI	*P* value ^a^
IBS	1.77	1.63–1.92	<.001
Age	1.00	1.00–1.00	0.018
Female sex	1.28	1.19–1.37	<.001
Insurance premium (NTD)			
>=40000	0.99	0.89–1.11	0.924
20000–39999	1.01	0.91–1.11	0.890
<20000	1.00		
Urbanization level			
Urban	1.02	0.90–1.16	0.718
Suburban	1.03	0.90–1.18	0.662
Rural	1.00		

HR: hazard ratio. CI: confidence interval.

^a^ Tested by multivariate Cox proportional hazard regression.

**Table 4:** IBS predicting risk of onset of microscopic colitis, IBD, and colorectal cancer.

Endpoint	HR	95% CI	*P* value ^a^^,^^b^
Microscopic colitis	1.72	1.58–1.87	<.001
Inflammatory bowel disease	1.92	1.49–2.48	<.001
Colorectal cancer	3.63	2.54–5.19	<.001

HR: hazard ratio. CI: confidence interval.

^a^ Tested by multivariate Cox proportional hazard regression.

^b^ Adjusted for age, sex, insurance amount, and urbanization level.

**Table 5:** IBS predicting microscopic colitis, IBD, and colorectal cancer stratified by sex.

Endpoint	HR	95% CI	*P* value ^a^^,^^b^
Women			
Microscopic colitis	1.66	1.49–1.86	<.001
Inflammatory bowel disease	1.71	1.22–2.41	0.002
Colorectal cancer	3.35	1.98–5.66	<.001
Men			
Microscopic colitis	1.79	1.57–2.04	<.001
Inflammatory bowel disease	2.27	1.53–3.35	<.001
Colorectal cancer	3.88	2.38–6.34	<.001

HR: hazard ratio. CI: confidence interval.

^a^ Tested by multivariate Cox proportional hazard regression.

^b^ Adjusted for age, insurance amount, and urbanization level.

### Rate of Colonoscopy and Sigmoidoscopy Use Before IBS Diagnosis

**Fig 2:**
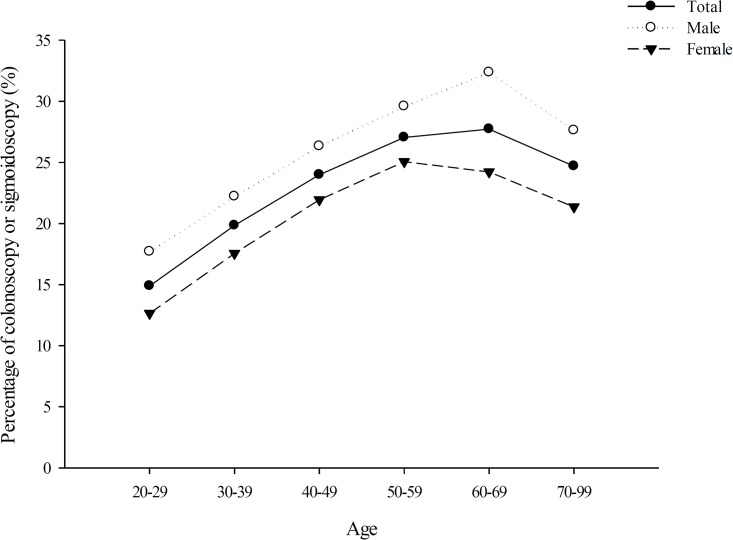
Percentage of patients with IBS receiving colonoscopy and sigmoidoscopy in Taiwan, stratified by sex and age.

Among patients diagnosed with IBS during at least 2 medical encounters, 23.04% (25.81% men and 20.65% women) had undergone colonoscopy and sigmoidoscopy within 2 years before the IBS diagnosis. As illustrated in Fig 2, men had a higher rate of colonoscopy and sigmoidoscopy use than women did across all age groups. The rate of colonoscopy and sigmoidoscopy use in patients with IBS gradually increased with age for both sexes and then decreased after 59 and 69 years of age in women and men, respectively.

## Discussion

During 2003–2013, the IBS incidence rates in men and women were estimated to be 48.90 and 53.51 per 10 000 person-years, respectively. The age-adjusted annual incidence rate of IBS decreased from 56.28 to 44.56 per 10 000 person-years from 2003 to 2013. A decreasing trend was observed in the IBS incidence rates over time; this may reflect an increase in the consensus regarding the diagnostic practices for IBS among clinicians in Taiwan during 2003–2013. In particular, the IBS incidence rates decreased considerably from 2007 to 2008. Coincidentally, the publication of the Rome III criteria was in 2006. Comparing the Rome II and Rome III adult diagnostic criteria for IBS revealed that a new requirement for the frequency of abdominal pain or discomfort per month (>3 d/mo) was stipulated in the Rome III criteria, which may clarify the sharp drop in the incidence rate from 2007 to 2008.

Women showed a slightly higher incidence of IBS than men did—the female-to-male ratio was 1.14:1. Notably, the female predominance in the incidence rate disappeared after the age of 69 years, and men in the 70–99 age group were associated with a higher incidence of IBS. These results indirectly and partially support the role of sex hormones in IBS development. The association between female hormonal status and IBS has been widely investigated. Exacerbation of bowel symptoms is associated with premenstruation and menstruation periods, during which estrogen and progesterone levels are low[9]. By contrast, a reduction in IBS incidence rates has been consistently reported across studies in postmenopausal women[9]. Although male sex hormones may protect against IBS development, studies on this topic are scant, with the reported results being inconsistent[8, 9]. The roles of female sex hormones in the development of IBS and of male sex hormones in protection against IBS remain unclear and warrant further investigation.

Although it is recommended that a diagnosis of IBS be made for patients who fulfil the symptom-based criteria with negative concerning features[4, 11], in practice, most clinicians diagnose IBS by excluding diagnoses of other organic diseases[11]. A review revealed IBS incidence rates to be lower in people aged older than 50 years than in those aged younger than 50 years[7]; by contrast, we observed that the IBS incidence rates increased with age among Taiwanese adults. Physicians in Taiwan are possibly more comfortable in relying solely on symptom-based criteria to diagnose IBS in the older population (aged ≥70 years) than in younger patients, in whom IBS is less likely to be diagnosed without colorectal cancer screening. Although women showed only slightly higher IBS incidence rates, men with IBS had considerably higher rates of endoscopy procedure use; this is a notable observation for clinicians, health educators, and policymakers in Taiwan. The sex differences in the rates of colonoscopy and sigmoidoscopy use in patients with IBS may simply represent disparities in health service use between men and women. This explanation is slightly inconsistent with the conventional assumption that women use more health services than men do[17]. The female sex increases physicians’ diagnostic uncertainty, thus reducing the likelihood of physicians ordering tests and medications appropriate for urgent cardiac conditions in female patients[18]. Hence, the sex differences in the rates of endoscopic procedure use before IBS diagnosis among patients with IBS may be attributed to physicians behaving differently when approaching men and women with colonic complaints in terms of diagnostic and treatment decisions, with the consideration that women have a higher IBS prevalence. Sex differences in diagnostic uncertainty and treatment decisions for patients evaluated for IBS warrant further investigation.

In the present study, we followed a group of patients with new onset IBS in 2003 and tracked the onset of selected colonic organic diseases over 10 years. We observed that for both men and women, the risk of subsequent onset of the composite outcome of organic diseases was 1.77 times higher in patients previously diagnosed with IBS than in their counterparts during the 10-year observation period (2003–2013). The risk of IBD, microscopic colitis, and colorectal cancer ranged from 1.7–3.6 times higher in patients with IBS compared with those without. Our findings are consistent with those of a cross-sectional study regarding the yield of colonoscopy in a cohort of patients fulfilling diagnostic criteria for IBS, reporting that among patients with symptoms compatible with IBS with or without concerning features, most patients exhibited organic colonic diseases following colonoscopic investigations[19]. Along with our findings, we conclude that IBS and other colonic organic diseases likely coexist and share common pathophysiological pathways; for instance, mechanisms of IBS and IBD overlap [20]. IBS may not be considered an inherent clinical entity; therefore, a more detailed clinical evaluation may be recommended as the most appropriate practice to exclude organic diseases in patients evaluated for IBS. However, a meta-analysis reported evidence questioning the significance of routine colonoscopy and biopsy for excluding microscopic colitis diagnosis in patients with typical IBS symptoms; the meta-analysis revealed that despite one-third of patients with microscopic colitis reporting symptoms compatible with IBS, the IBS prevalence and the likelihood of microscopic colitis were not higher than those in other patients with diarrhea[21]. This phenomenon warrants further research.

The strengths of our study are that we analyzed claims data of a representative sample (*N* = 1 million) randomly selected from the beneficiaries of the Taiwan National Health Insurance program, which covers more than 99% of the residents of Taiwan. We also used a strict case ascertainment process to identify patients with IBS, in addition to adjusting socioeconomic confounders in the models. Nevertheless, one major limitation of our study is that information associated with diagnostic decisions regarding IBS could not be obtained from the claims database. Nonetheless, all insurance claims of the National Health Insurance program are routinely scrutinized by medical reimbursement specialists and are subject to peer review. The institutions are subject to penalties and no reimbursement if the coding of diseases or treatments is incorrect. As such, coding errors are minimized to a lower degree. For case ascertainment, we restricted our IBS cases to those with at least 2 medical encounters with claims records corresponding to ICD-9-CM codes of IBS within 1 year. Finally, it must be born in mind that as the data being studied was based on a health insurance database, the trend may reflect IBS-associated health care utilization rather than the actual disease incidence.

In conclusion, the incidence rates of IBS increased with age and were slightly higher in women than in men among Taiwanese adults. During 2003–2013, IBS incidence rates gradually decreased over time. The sex differences in IBS incidence and variations of clinical decision making between sexes in patients with IBS warrant further research. Both male and female patients with IBS are associated with an increased likelihood of future onset of IBD, microscopic colitis, and colorectal cancer.
